# The Role of the Periaqueductal Gray Matter in Lower Urinary Tract Function

**DOI:** 10.1007/s12035-018-1131-8

**Published:** 2018-05-26

**Authors:** Aryo Zare, Ali Jahanshahi, Mohammad-Sajjad Rahnama’i, Sandra Schipper, Gommert A. van Koeveringe

**Affiliations:** 1 Department of Urology, Maastricht University Medical Center, Maastricht, The Netherlands; 2 School for Mental Health and Neuroscience, Faculty of Health, Medicine and Life Science, Maastricht, The Netherlands; 3 Department of Neurosurgery, Maastricht University Medical Center, Maastricht, The Netherlands

**Keywords:** Periaqueductal gray matter, Bladder, Incontinence, Micturition, Brain

## Abstract

The periaqueductal gray matter (PAG), as one of the mostly preserved evolutionary components of the brain, is an axial structure modulating various important functions of the organism, including autonomic, behavioral, pain, and micturition control. It has a critical role in urinary bladder physiology, with respect to storage and voiding of urine. The PAG has a columnar composition and has extensive connections with its cranially and caudally located components of the central nervous system (CNS). The PAG serves as the control tower of the detrusor and sphincter contractions. It serves as a bridge between the evolutionary higher decision-making brain centers and the lower centers responsible for reflexive micturition. Glutamatergic cells are the main operational neurons in the vlPAG, responsible for the reception and relay of the signals emerging from the bladder, to related brain centers. Functional imaging studies made it possible to clarify the activity of the PAG in voiding and filling phases of micturition, and its connections with various brain centers in living humans. The PAG may be affected in a wide spectrum of disorders, including multiple sclerosis (MS), migraine, stroke, Wernicke’s encephalopathy, and idiopathic normal pressure hydrocephalus, all of which may have voiding dysfunction or incontinence, in certain stages of the disease. This emphasizes the importance of this structure for the basic understanding of voiding and storage disorders and makes it a potential candidate for diagnostic and therapeutic interventions.

## Introduction

The PAG is the central gray matter of the midbrain, in continuance with the circumventricular organs, and is to a large extent analogous to the gray matter of the spinal cord. Due to its axial location, the PAG is involved in various important functions, including autonomic [1, 2], behavioral [3], pain [4], and micturition control. The role of the PAG in the control of the bladder function encompasses both downstream connections, as well as connections with the higher brain centers involved in decision-making. Barrington was the first one to discover the function of the pontine micturition center (PMC) (Barrington’s nucleus), and the role of the midbrain in the control of micturition, though he did not mention the PAG [5].

**Fig. 1:**
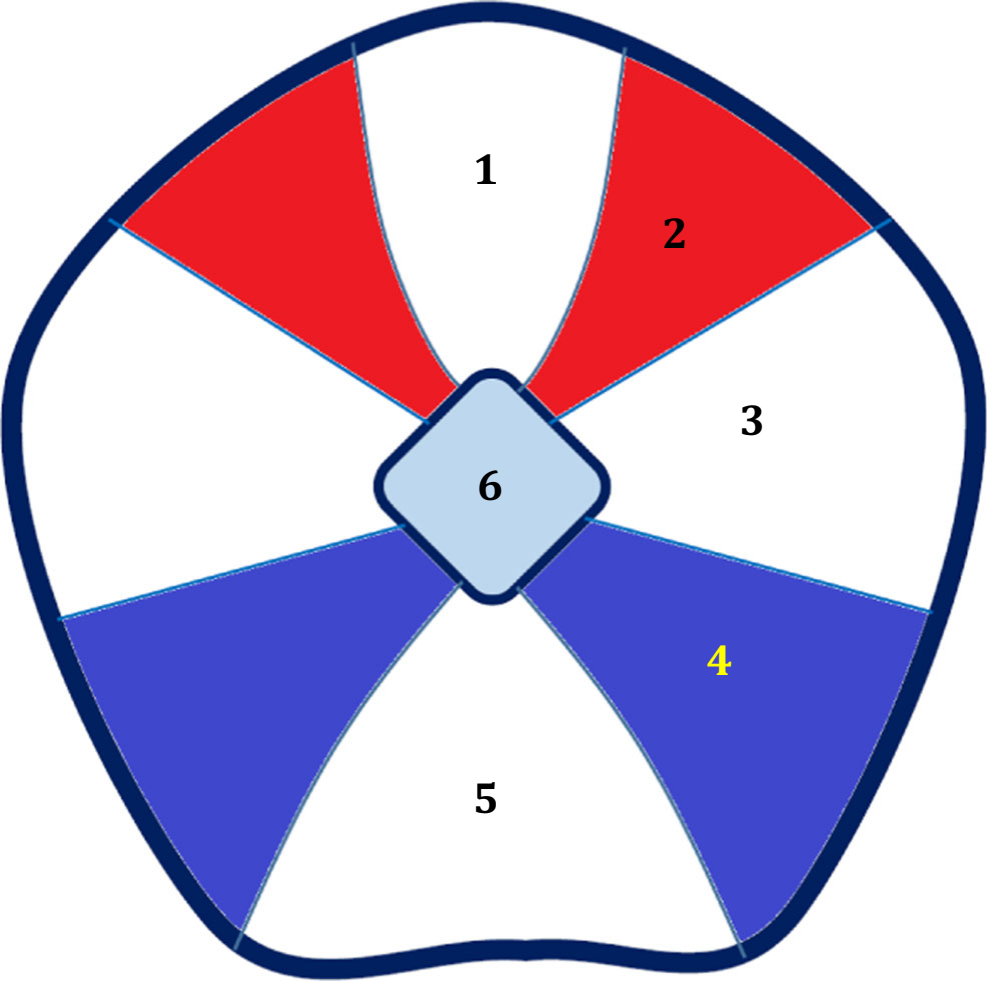
Schematic representation of a coronal section through the caudal part of the rat PAG, showing columnar segmentations. Two pairs of columns with major functional contribution in micturition, namely dorsolateral and ventrolateral, are highlighted in red and blue, respectively. 1. Dorsomedial column. 2. Dorsolateral column (red). 3. Lateral column. 4. Ventrolateral column (blue). 5. Area of dorsal raphe and some cranial nerve nuclei. 6. Central aqueduct

The PAG has been found to bear functionally separate columns [6], which are also different histopathologically in the human brain [7]. There are three pairs of columns, namely ventrolateral (vlPAG), lateral (lPAG), and dorsolateral (dlPAG), as well as a single dorsomedial column (dmPAG), in the PAG (Fig. 1). These columns can be functionally divided into two groups, having opposite autonomic functions: the ventrolateral column, which has parasympathetic functions, and the lateral and dorsolateral columns which have sympathetic functions [8, 9]. Even considering the amount of expression of specific markers, or distant connections, there exist two distinct components: the dorsolateral pair of columns, and the remaining columns, including the ventrolateral, lateral, and dorsomedial pairs [10]. dlPAG is functionally more closely related to the midbrain tectum, and the remaining columns may be considered more closely related to the periventricular structures [10]. All of the PAG columns have ipsilateral and contralateral reciprocal connections [11]. Internal connections also exist within each column [11]. Below, we will discuss the role of the PAG in the control of micturition.

## Connections of the PAG

**Fig. 2:**
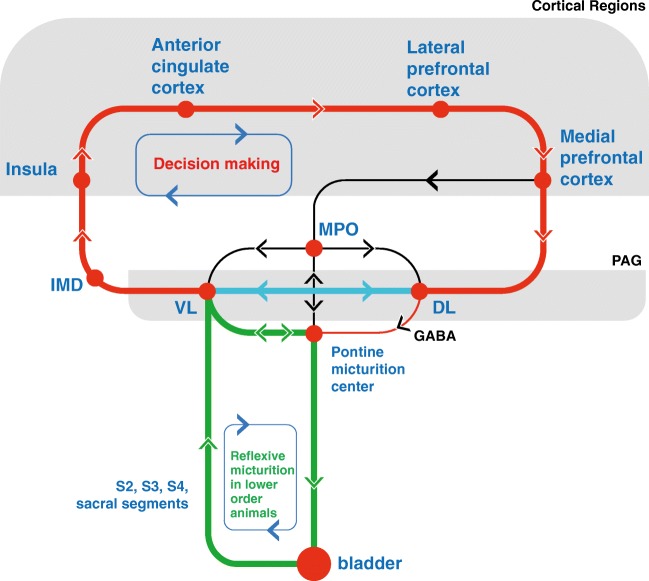
Major central pathways of micturition. The bladder distension signals first reach the ventrolateral column of the PAG [12, 13]. This may trigger the PMC and induce the contraction of the bladder without any interference from the higher brain centers, which may be the underlying reason of infantile incontinence, or the often-reflexive micturition in rats. The thalamus does not receive direct signals from the bladder or the spinal cord. Instead, these sensory signals must first synapse in the PAG. The vlPAG projects to the intermediodorsal nucleus of the thalamus, and then the insula, in rat [14]. These signals finally reach the medial prefrontal cortex [15]. The projections from medial prefrontal areas predominantly reach the dorsolateral PAG columns [16]. It is noteworthy to mention that the existence of the prefrontal cortex in rodents is controversial. The involvement of the medial preoptic area [17, 18] may provide additional safe signaling for the start of voiding, or may even serve as a coordinating center to harmonize the voiding with mating or territorial demarcation behavior [19]. There is a profound network of intercolumnar connections in the PAG [11] which bind the cortical and peripheral feedback loops and provide continued processing of the incoming signals of the level of the bladder fullness, monitoring the environmental states, and decision for the feasible opportunity to void. The brain pathways have been shown by various circuits [15, 20–22] according to characteristics found in functional imaging. Note the decision-making and reflexive micturition feedback loops, active in higher- and lower-order animals respectively. VL ventrolateral column, DL dorsolateral column, IMD intermediodorsal nucleus of the rat thalamus (analogous to mediodorsal nuclei of the human thalamus), MPO medial preoptic area of hypothalamus

The PAG has extensive connections with the cortex (prefrontal, cingulate, and insular gyri), diencephalon (thalamus and medial preoptic area of hypothalamus (MPO)), brainstem (PMC), and the spinal cord (sacral segments) (Fig. 2) [15, 20–25]. Caudal connections of the PAG have been investigated by tracing techniques, which are briefly mentioned in this section. The cranial connections with cortical regions have been discovered mainly by connectivity analysis in functional imaging studies, which will be described in the following sections.

PMC, as another important micturition control center, has bilateral connections with vlPAG [23, 24, 26], as well as receiving afferents from dlPAG and the MPO [23, 27, 28] (Fig. 2). In parallel, MPO has direct connections with the PAG [29–31]. The MPO, rich in androgen and estrogen receptors, is integral to the limbic, or the so-called emotional motor system, regulating sexual behavior. Since micturition is an important behavioral signal in animal territorial demarcation, and the scent of urine may additionally serve as a sexual message, the PAG may integrate the micturition and mating functions.

The dlPAG along with vlPAG receive afferents from spinal parasympathetic and dorsal commissural nuclei of the lumbosacral cord [12, 32, 33], which have ipsilateral dominance [32]. This pathway contributes to the awareness of the PAG to the state of the bladder fullness. Efferent PAG connections project indirectly via PMC to the spinal segments and are distributed via somatic or autonomic nerves and ganglia to the detrusor muscle or sphincters [34].

### The vlPAG as the Major PAG Column with Connections to the Bladder

The vlPAG has direct connections with the spinal segments [35]. It is also the main column receiving afferents from the lumbosacral cord, which have ipsilateral dominance [32] and originate from laminae V, VII, and VIII [35]. Spinal neurons throughout the cord projected more to the lPAG and the vlPAG rather than to the dlPAG or the dmPAG, and specially segments from the S1-S3 projected to the central portion of the lPAG and the vlPAG [13]. Indeed, only few neurons, mainly located in the lumbosacral segments, project to the dorsomedial and dorsolateral PAG columns [36]. The quantity of projections weighs toward the ventrolateral column, more than to the other columns, and there exists evidence showing the higher significance of the role of the vlPAG, regarding the control of bladder function.

Further evidence regarding the importance of vlPAG in micturition control is as follows. Most c-Fos reactivity is induced in the vlPAG after chemical irritation [37] or electrical stimulation (unpublished data) of the bladder. c-Fos is a transcription factor expressed after neuronal activation. Electrical stimulation of the pelvic nerves in the cat evoked maximum field potentials in vlPAG [38]. Studies in cats confirm that it is the vlPAG, of which the electrical or chemical (DL-homocysteine) stimulation results in the contraction of the bladder [24]. Chemical stimulation of the vlPAG by D,L-homocysteic acid (DLH) in rats increases the frequency of micturition [39]. On the other hand, stereotaxic injection of the inhibitory mediator cobalt chloride into the caudal vlPAG reversibly attenuates bladder contractions and external urethral sphincter (EUS) electromyographic activity in rats [40]. Injection of other inhibitory or stimulatory agents, CoCl2 and L-glutamate, into the vlPAG leads to suppression or stimulation of voiding in rats, respectively [41]. Taking these studies into account, it is most likely that the vlPAG would be the primary station of the ascending PAG afferents, and it may secondarily relay these signals to the other PAG columns [11].

## PAG and the Function of the Bladder

Functional studies regarding the control of the PAG over the bladder encompass electrical or chemical stimulation of the PAG, single-unit recordings in the PAG, or indirect evaluation of the function by measuring the neuronal markers c-Fos or nerve growth factor (NGF). We can differentiate two major columns, the ventrolateral and the dorsolateral columns, for playing the main role in the micturition-related PAG functions.

### Studies Featuring the Function of the Ventrolateral Column of PAG

Some studies have a top-down design, in such a way that central electro-stimulation at PAG is accompanied by peripheral evaluation of function at bladder level. Electrical stimulation of the vlPAG elicits either contraction or inhibition of the bladder [42]. The optimum sites for evoking bladder contractions were located in and close to the laterodorsal tegmental nucleus (LDT) (which is the same structure functionally called PMC), and in the PAG, just dorsal or dorsolateral to the LDT, in rats [42]. Electrical stimulation of the ventral PAG elicits neuronal firing at the postganglionic nerves of the bladder [43]. Studies in cats confirm that electrical or chemical (DL-homocysteine) stimulation of vlPAG results in contraction of the bladder [24].

Inversely, some other studies have a down-top design, i.e., peripheral electro-stimulation is accompanied by central evaluation. Electrical stimulation of the pelvic nerves in the cat evokes maximum field potentials in vlPAG [38]. Since the regions activated by the pelvic nerve stimulation differ from those activated by stimulation of the sensory pudendal or superficial perineal nerves, it is possible that specific pathways exist for different nerve inputs to the PAG [38]. However, in another study, electrical stimulation of afferents in the pelvic nerve of the rat evoked field potentials in the dorsal part of the PAG [43].

Single-unit recordings in PAG columns either with or without stimulation of the bladder derived some information regarding the PAG neuronal activity. The vlPAG shows three different patterns of neuronal firing rate during the micturition: increased firing rate, decreased firing rate, or no correlation with the micturition [44]. These neurons may correspond to specific functions they have during the micturition, with some of them contracting the detrusor, some relaxing the sphincter, some receiving afferent sensory signals, and others unrelated to the micturition. A similar study accompanied by intravesical pressure recordings was conducted in cats [45]. However, only 16 out of 84 neurons that were recorded were located in the PAG. The rest of the neurons were in adjacent midbrain areas. Nevertheless, this study emphasizes the existence of neurons in ventrolateral and lateral columns of the cat PAG, with firing patterns which change, corresponding to specific phases of micturition [45].

Chemical stimulation or suppression of the PAG exerts similar effects over the bladder. As detailed above, chemical stimulation of vlPAG increases the frequency of micturition in rats [39, 41] and contracts the detrusor muscle in cats [24]. On the other hand, chemical inhibition of vlPAG attenuates the detrusor and EUS contractions in rats [40, 41]. Similarly, bilateral electrolytic lesion of vlPAG and lPAG in cat attenuates detrusor contractions [45].

Neuronal activation may also be evaluated by specific markers such as c-Fos or NGF expression. Either anatomical manipulation or chemical irritation of the bladder may induce increased neuronal activity in the PAG. Increased c-Fos [46] or NGF [47] reactivity in vlPAG was observed after the induction of stress urinary incontinence by transabdominal urethrolysis in rats. Chemical bladder irritation, which is a painful stimulus, induced c-Fos expression in the PAG, though the exact PAG column was not stated [48].

### Studies Featuring the Function of the Dorsolateral Column of PAG

Some studies proposed the possibility of the existence of a specific micturition-suppressing region in dlPAG, acting via GABAergic inhibition of PMC [49]. Electrical stimulation of dlPAG elicits various types of reactions from the bladder. Electrical stimulation of the dorsal part of the PAG, including the dorsomedial and the dorsolateral columns, in rats, resulted a higher frequency of voiding, as well as some behavioral responses such as tense immobility, accompanied by exophthalmos and running and jumping responses [50]. A similar result, as bladder contraction, was encountered after electrical stimulation of points concentrated at the superior collicular and intercollicular levels, in an area involving the deep layers of the superior colliculus, the dlPAG, and the tegmental reticular formation, neighboring the most lateral border of the PAG, in cat [51]. Nevertheless, this may be an erroneous conclusion due to inadvertent diffusion of electrical current to other PAG columns. In fact, electrical stimulation or glutamate microinjection within either ventrolateral, lateral, or dorsolateral columns evoked the rise of the intravesical pressure, as well as an increase in blood pressure [52]. Another reason behind this discrepancy between different studies may be the difference in cranio-caudal location of the stimulation sites. For example, a similar study in cat mentioned rostral part of dorsal PAG and caudal part of ventral PAG to be the main sites, at which high-frequency electro-stimulation causes inhibition of micturition [45].

### Other Functional Studies

Some other studies show that stimulation of either the vl- or dlPAG would suppress the contraction of the bladder. Deep brain stimulation (DBS) in vlPAG attenuates or completely suppresses the voiding in rats and humans [53]. Similar physiological activities such as rhythmic straining reflexes or defecation, alongside with micturition, are inhibited by electrical stimulation of the ventral or dorsal PAG, dorsal raphe nucleus, and central tegmental field, with similar threshold intensities, in dogs [54]. These studies show that the electrical stimulation of various midbrain structures, and not only specific PAG columns, may suppress some pelvic functions. Such equivocal results may be due to a possible jamming effect over the normal electrical circuitry, contributing to the negative impact of DBS on micturition [39]. One possible explanation is the disruption of the normal coordinated voiding activity in the detrusor and sphincter muscles, after DBS.

Since the micturition reflex is under unconscious control during sleep, and nocturnal enuresis is a common associated disorder, it would be interesting to investigate the function of PAG during sleep, and the associated changes in detrusor contractions. Simultaneous recordings of the detrusor pressure, EUS electromyogram (EMG), cortical electroencephalogram (EEG), and single-unit activity in the PAG in rats reveal that during slow-wave EEG activity (SWA), voiding becomes more irregular and detrusor voiding pressure threshold and voiding volume threshold, and the duration of the bursting activity in the EUS EMG is raised, all in line with maintaining continence during sleep [55]. SWA is associated with slower neuronal firing rate in the PAG as well. Different sleep-like brain states are associated with changes in urodynamic properties, suggesting changing excitability of the micturition circuitry in the PAG. This may uncover some underlying factors in the pathophysiology of nocturnal enuresis [55].

In summary, the PAG receives ascending sensory signals from the bladder and can modulate the bladder function by its descending efferent connections. Such bilateral connections may be important in conducting reflexive micturition in rodents, or the immature human. This comprises a feedback loop, with PAG continuously monitoring the state of bladder fullness, and induction of voiding in a suprathreshold-filled bladder (Fig. 2).

## The Role of Distinct Cell Groups in PAG Function

To better understand the organization of neural circuits, different cell populations contributing to various neural pathways are investigated (Table 1). The PAG, like most other brain regions, has a variety of distinct cell groups and has immense connections with its cranially and caudally located CNS structures, related to the control of the micturition. Among these cell groups are dopaminergic, serotoninergic, glutamatergic, and GABAergic neurons, and cells expressing neuronal nitric oxide synthase (nNOS). Dopamine, serotonin, and glutamate are stimulatory neurotransmitters, and GABA and nNOS are inhibitory neurotransmitters. PAG neurons express receptors for all of these neurotransmitters (Table 1).

**Table 1:** Synthesis of neurotransmitters and the expression of their receptors in the PAG, and their functional significance

	Synthesis^1^	Receptor^1^	Role in micturition (all experiments were performed on rats)
Dopamine	✓ [56]	✓ [57]	Inhibitory: ■ Application of a D1 receptor antagonist into the PAG facilitated the micturition reflex [58].
Serotonin	✓ [59]	✓ [60]	No role detected so far: ■ It seems to be responsible for nociception in the PAG [61].
Glutamate	✓ [62]	✓ [63]	Excitatory: ■ Chemogenetic or optogenetic stimulation of vlPAG glutamatergic neurons leads to voiding and detrusor contraction [64]. ■ Glutamatergic vlPAG cells were activated after bladder electro-stimulation [65]. ■ Glutamate microinjection within the PAG evoked a rise of intravesical pressure [41, 52]. ■ Saline infusion into the bladder with consequent induction of the micturition reflex resulted in increased extracellular glutamate levels in the PAG [61].
GABA	✓ [66, 67]	✓ [68, 69]	Inhibitory: ■ Chemogenetic or optogenetic activation of vlPAG GABAergic neurons delays detrusor contraction and inhibits voiding [64]. ■ Microinjection of a GABA agonist into the vlPAG of the rat depressed reflex voiding frequency, whereas microinjection of a GABA antagonist into the same region increased reflex voiding frequency [70].
Opioid	✓ [71]	✓ [72]	Inhibitory: ■ Injection of a μ receptor agonist into the caudal vlPAG abolished volume-evoked micturition [73]. ■ Intracerebroventricular injection of morphine or a μ agonist showed consistent inhibition of spontaneous urinary bladder contractions [74].

The check (✓) mark positively denotes that the chemical mediator identified is either synthesized in the PAG, or the PAG bears receptors for them, in conjunction with their respective references

^1^Capability to synthesize and the bearing of specific receptors for a particular neurotransmitter imply the existence of efferent or afferent pathways incorporating that particular neurotransmitter in the PAG

The vlPAG has distinct groups of glutamatergic cells which can stimulate other centers [62]. Chemogenetic or optogenetic stimulation of glutamatergic neurons in the vlPAG leads to detrusor contraction and voiding [64]. By contrast, chemogenetic or optogenetic activation of vlPAG GABAergic neurons delayed detrusor contraction and inhibited voiding [64]. The vlPAG GABAergic cells stimulated in this experiment were most probably interneurons [75]. On the other hand, the main inhibitory GABAergic input to the vlPAG, relevant to the micturition, projects from dlPAG [49]. Glutamatergic cells of the vlPAG also control other important functions, including freezing [75] and nociception [76], which are controlled by two separate cell groups in the vlPAG [75]. Whether these cell groups are different from those vlPAG glutamatergic cells controlling voiding, or have some overlap, remains to be elucidated.

### Excitatory Signaling

In this section, we mention glutamatergic, dopaminergic, and serotoninergic neuronal signaling, arising from PAG and influencing micturition.

Glutamate serves as an excitatory neurotransmitter [77]. Its extracellular levels have been shown to be increased in PAG, after bladder distension or voiding, by microdialysis studies [58, 61]. Glutamatergic cells may project back to brainstem structures related to the control of the bladder contraction, to fulfill a micturition reflex, or to higher cortical regions to undergo further analysis and decision-making. The vlPAG receives afferents from the lumbosacral cord [32] and has direct connections with the PMC [32], which then controls the micturition by its efferents to sacral parasympathetic segments. This circuit is especially very active in rodents and probably in the immature human infant, while higher decision-making brain centers are still underdeveloped. vlPAG glutamatergic cells probably stimulate the PMC, after receiving suprathreshold sensory signals from a full bladder. As a clinical correlate, multiple system atrophy (MSA), which is an extrapyramidal disease, has micturition symptoms including frequency, urgency, incontinence, or incomplete bladder emptying, as part of its manifestation. The number of glutamatergic cells in the ventrolateral, lateral, dorsomedial, and to a lesser extent dorsolateral PAG columns was shown to be decreased in MSA [78].

The role of dopaminergic neurotransmission over micturition is controversial. Microinjection of SCH-23390, a dopamine antagonist, into the PAG, had contrary results over micturition in two different studies [58, 79]. The PAG dopaminergic neurons mostly project rostrally to higher brain regions [80, 81]. There is a loss of putative wake-active PAG dopaminergic neurons in patients suffering from either MSA or dementia with Lewy bodies, which may contribute to excessive daytime sleepiness in these disorders [82]. Both of these conditions have micturition problems. As micturition is in coordination with the sleep-wake cycle, their possible role on micturition must be indirect, via influence over other autonomic systems.

Serotoninergic cells are abundant in the brain. The evidence regarding the role of serotonin in the regulation of micturition is very limited. Few serotoninergic neurons are present in the PAG, which are particularly scattered in vlAPG, close to the dorsal raphe nuclei. This actually makes these few cell populations suspicious to be extra-raphe serotoninergic cells, not being part of the main PAG columns. There is not much known about the micturition-related pathways within the PAG, involving serotonin for neurotransmission. The vlPAG serotoninergic cells inhibit the ejaculation in rats and may contribute to SSRI (selective serotonin reuptake inhibitor)-induced inhibition of ejaculation [83]. Yet, regulation of the autonomic function of the genital organs is different from that of the bladder. Since glutamate modulates 5-HT release in the PAG [84], and serotoninergic cells of the PAG also have projections toward other brainstem regions [85], there may be an indirect relation between the bladder stimulation and the serotonin system.

### Inhibitory Signaling

Inhibitory signaling from PAG arises from GABAergic cells, or neurons expressing nNOS.

GABAergic transmission has an indirect role over vlPAG, by internal connections via the dorsolateral column of the PAG [11]. GABAergic cells of dlPAG project to vlPAG to suppress the micturition reflex [49]. PMC has reciprocal connections with the vlPAG [23, 24] and receives inhibitory GABAergic input from dlPAG [49] (Fig. 2). The existence of reciprocal connections between various columns of the PAG [11] emphasizes the existence of a micturition-suppressing region in dlPAG.

The bladder’s intramural ganglia, dorsal root ganglia, and spinal cord contain nitric oxide (NO), the expression of which shows plasticity, following pathological lesions, such as pelvic nerve injury, chronic bladder irritation, and urethral obstruction [86]. The rate of NO production in brain is dependent on dynamic regulation of its synthetic enzyme, nNOS [87]. There has been no report about the role of nNOS in the brain, related to the physiology of micturition. Most functions mediated by nNOS, including modulation of the cardiovascular, behavioral, or nociceptive functions, have been found to be mainly active at dorsal PAG [88–90]. The existence of any possible role over the micturition pathways in the PAG by NO would probably be mediated by its interactions with GABA [91]. Its inhibitory neuronal activity within the PAG [92] may also be explained accordingly, since GABA is an inhibitory neurotransmitter.

An overall comparison of various cell groups in a standardized setup shows that in contrast to serotonergic, dopaminergic, GABAergic, and nNOS-synthesizing cells, only vlPAG glutamatergic neurons are activated upon receiving afferent bladder sensory signals [65].

Table 1 summarizes some key cell types in the PAG, bearing particular neurotransmitters, and their corresponding receptors, and describes their functions in micturition.

### Other Important Chemical Mediators of PAG Function

Since the PAG is also a center for pain control, endogenous opioids have significant presence in this region. Their potential engagement in the control of voiding has been investigated in various ways. Among the different types of opioid receptors and PAG columns, it is only μ (mu) receptors which have an inhibitory effect on the vlPAG, by abolishing volume-evoked micturition [73]. Intracerebroventricular injection of morphine or the μ agonist morphiceptin confirms the aforementioned findings, showing consistent inhibition of spontaneous urinary bladder contractions [74]. The rapid onset of action and its limited distribution, shown by the intraventricular dye injection studies, indicates that its actions are confined predominantly to the periventricular and periaqueductal or associated areas, and not to the spinal cord [74].

The α1-adrenergic receptor antagonist tamsulosin, and the PDE-5 inhibitor sildenafil, significantly suppressed the increase in neuronal activities measured by the expression of c-Fos and NGF, in the vlPAG, in an overactive bladder rat model [93]. Further evidence for the role of phosphodiesterase system comes from studies using caffeine. Caffeine is a methylxanthine alkaloid chemically related to the adenine and guanine bases of DNA. Its mechanism of action is by phosphodiesterase inhibition and adenosine antagonism. Caffeine administration to rats for 14 days increased bladder smooth muscle contraction pressure and time, determined by cystometry [94]. Expression levels of c-Fos and NGF in the vlPAG were also significantly increased following the administration of caffeine [94]. Hence, the phosphodiesterase system has possible regulatory role over the PAG and can influence the micturition.

## Functional Imaging of the PAG

**Fig. 3:**
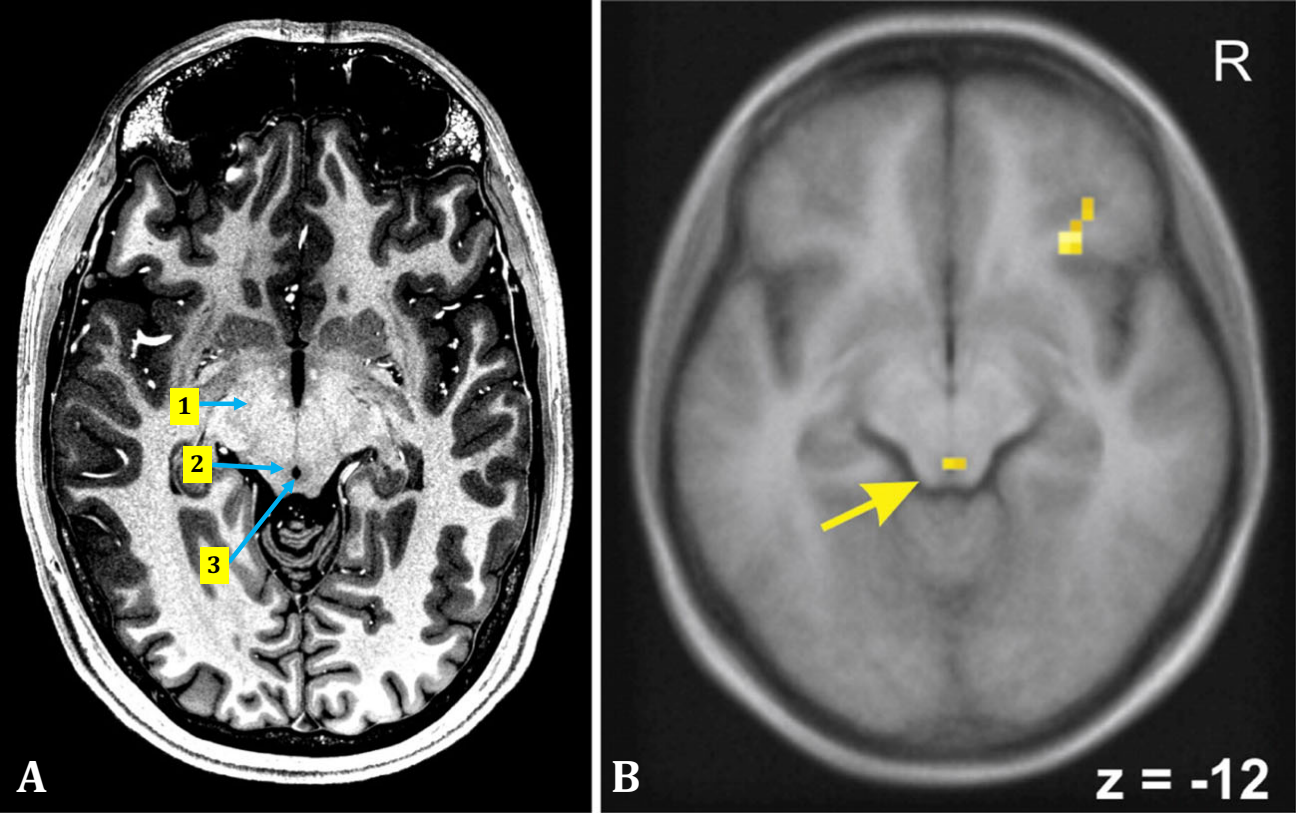
**a** A transverse section through the human midbrain in a normal subject, showing the cerebral peduncles (1), the PAG (2), and the aqueduct (3) (7 Tesla MRI) [95, 96]. **b** An fMRI showing the activated regions in a transverse section of the human brain. The PAG (arrow) along with the right insula shows more activity in a full bladder rather than in an empty bladder, during attempted micturition (1.5 Tesla fMRI) (reprinted with permission from Elsevier) [97]

Functional imaging is a non-invasive tool for visualizing the activation of specific brain regions in response to various stimuli, respecting sensory afferent and motor efferent functioning. However, state-of-the-art functional imaging techniques usually lack adequate spatial resolution to make reliable statements about the function of a specific column of the PAG (Fig. 3a, b). Nevertheless, functional imaging studies have shed light on different connections of the PAG (Figs. 2 and 3b). They can also detect defects in some structural or functional pathologies involving the PAG. Functional MRI (fMRI), as the most common type of functional imaging, is the imaging modality widely used to determine the activated brain regions in different phases of micturition.

Since PAG is also a center for handling nociceptive signals, it would be interesting to differentiate pain from other afferent sensory information processing. Both distention of the bladder and painful stimuli may activate the PAG, with different patterns. The vlPAG, among other regions, was activated in human PET scans after bladder distension, but not with intravesical ice bladder instillation, revealing different pathways in bladder distension and pain [98]. fMRI on healthy females shows that cold (pain) sensation is processed differently from bladder distension at the supraspinal level [99]. Parallel in vivo studies in mice revealed that selective optogenetic activation of bladder sensory fibers can differentially modulate nociceptive information and autonomic reflexes [100]. These findings show that bladder nociceptive and mechanosensory signals have separate pathways, both in the periphery and in the brain.

PAG is also activated in some similarly related physiological processes, like pelvic muscle contraction or rectal distention. In a non-voiding model of voluntary micturition control, ventral pons and the PAG showed more enhanced activation patterns by fMRI, in voluntary contraction than in the relaxation of the pelvic floor muscles [101]. Rectal distention in humans led to PAG activation detected by PET that was also associated with increased heart rate and with increased plasma adrenaline [102].

The PAG is active in both storage and voiding phases of the micturition cycle, but the extent of its activity differs between these two phases. During the storage phase, the PAG is activated, but the PMC is inactive, and during the voiding, the PMC maintains activation, and the activation of the PAG enhances [103].

Hereby, more evidence is provided supporting the activity of the PAG in storage and voiding phases of micturition.

### The PAG Activation During the Storage Phase:

These experiments are usually designed in such a way that the brain would be scanned, while the bladder is being passively filled by intravesical infusion of saline. This way, mechanosensory signals arising from an expanding bladder would reach central micturition control centers. The activity in the right anterior insula and the PAG in human was enhanced at higher bladder volumes, in a non-voiding bladder, detected by fMRI [97] (Fig. 3b) and PET [104]. PAG activation after bladder filling is accompanied by the activation of the inferior parietal lobule, as well as the right insula and the dorsal anterior cingulate cortex (ACC) [105]. Passive filling and emptying of the bladder induce PAG activation as well [17, 106, 107]. Studies on Parkinson’s disease (PD) patients show that the activity of the PAG is enhanced in a full bladder, compared to an empty bladder [108]. Furthermore, DBS of subthalamic nucleus increases the PAG activity in PD patients [108]. This would indirectly influence the activity of other cortical regions related to micturition and ultimately restore afferent bladder information processing [108]. These findings point to the fact that the PAG processes the sensory signals derived from a filling bladder. These signals may then flow to specific cortical regions and reach the awareness.

### The PAG Activation During the Voiding Phase:

#### Initiation of Voiding

There is a special role for the PAG in the initial moments of the bladder emptying. The PAG is consistently active during “attempted micturition” [97] (Fig. 3b). During this maneuver, the individuals direct their attention to the sensations arising from the bladder and urethra and increase the desire to void as if initiating to allow the urine to pass [97]. Initiation of voiding induced significant activity in cortical regions, in addition to the PAG, as shown by fMRI, whereas actual micturition was associated with significantly less such activity [97]. Unsuccessful attempts at micturition result from inefficient activation of the PAG and PMC during the initiation phase, which itself may be secondary to inadequate antecedent activation of frontoparietal and cingulate cortices, involved in decision-making for the micturition behavior [109]. The same results can be found by PET scans [110]. fMRI studies found out that imitation or interruption of voiding by relaxation or contraction of the pelvic floor muscles in healthy women could induce activation patterns in the PAG, cortical regions, and some other micturition control centers [111]. The above findings emphasize the role of the PAG in the integration and relay of information coming from different areas (such as the spinal cord and the cortico-limbic system), which are essential in micturition physiology. This signifies the role of the PAG to be the trigger for implementing the decision for voiding.

Analysis of reflexive micturition in healthy rats by animal fMRI showed the activation of the PAG [112]. High-resolution animal fMRI in rabbit could specifically identify the vlPAG for having an important role in bladder dysfunction biofeedback [113]. This is confirmed by a PET study in humans also showing the vlPAG to be activated after distention of the bladder [98]. Columnar differentiation in human PAG could also be done by a PET scan, which identified the activation of the tegmental part of the PAG, denoting either ventrolateral or lateral columns, during micturition [114]. Nonspecific involvement of the PAG was mentioned in some other human PET scans [18, 115]. These discoveries are corroborated by a single-photon emission computed tomography (SPECT) in human, showing increased PAG activation during voiding [116].

With upcoming research by modern 7 Tesla and 9 Tesla MRI machines, we shall gain more precise information regarding the connections of individual PAG columns with specific parts of the brain.

#### Connectivity Analysis

It is often helpful to investigate the connections between one particular structure and other brain regions, and their temporal pattern of activation. The PAG has been found to be functionally connected with some other brain regions during voiding (Fig. 2). The physiophysiological interaction (PPI) is a sophisticated tool used to elucidate the effective connectivity between different brain regions and a particular task, i.e., the micturition. It is generally based on observing alterations in the slope of the regression line of two different brain regions, with respect to another region, or a particular task [117]. This method aids in revealing the cortical pathways, together with their associated functions (Fig. 2).

fMRI signals of the right insula/operculum change in parallel with PAG activity (indicating connectivity), when the subject perceives an enhanced urge to void during “attempted micturition” [97]. Indeed, positive functional connectivity exists between the vlPAG and brain centers involved in micturition, such as the ACC or insula, shown by resting-state fMRI (rsfMRI) [118]. The PAG clearly shows some laterality in its effective connectivity, which is to right insula during the filling of the bladder [119, 120].

White matter hyperintensities (WMH) are common in the older population and have been linked to demyelination, spongiosis, and glial proliferation, presumably after lacunar ischemic infarcts. Global WMH has been linked to impairments of mobility, cognition, affect, and continence [121]. These hyperintensities could be located in any of the central micturition control centers and are particularly related to the severity of the incontinence [122]. The PAG is one of the regions, of which activity shows positive correlation with the global WMH [123]. Hence PAG may be secondarily affected by WMH, contributing to the micturition symptoms.

The imaging modalities explained above may be used for experimental or diagnostic purposes. Putting various cortical connections of the PAG together, we find a circuit of continuous monitoring of the level of the bladder fullness, vigilance of the environmental circumstances, and deciding to void at the right moment. This comprises a cortical feedback loop, completed by incorporating the ventrolateral and dorsolateral PAG columns, for thinking and implementation of the decisions (Fig. 2).

## PAG in Neuropathological Conditions and Their Effects on Micturition

The PAG may be affected in several pathological states, and its role has been investigated in various disorders showing lower urinary tract problems as part of their spectrum of symptoms. Here, we explain some structural and functional disorders, affecting the PAG and compromising micturition.

### Structural Pathologies

The so far reported structural pathologies include stroke, neoplastic lesions, hydrocephalus, and spinal cord injury. Spinal cord injury would secondarily alter PAG activity states, as discussed below.

Structural damage involving the PAG has been reported to be a cause of voiding problems. Cerebral stroke, which is a debilitating disorder in the elderly, may cause voiding dysfunction. Urinary retention was among the manifestations of a patient suffering from acute ischemic stroke of the brainstem [124]. Diffusion-weighted MRI showed hyperintensity over the left paramedian segment of the lower midbrain, which includes the PAG. The computed tomography also showed a hemorrhagic transformation and intraventricular hemorrhage in the fourth ventricle [124], which is directly beneath the aqueduct. Either indirect ischemic damage of the PAG or chemical irritation by a hemorrhagic cerebrospinal fluid (CSF) inside the aqueduct could explain the micturition-related symptomatology. In an intracerebral hemorrhage (ICH)-induced rat model of neurogenic voiding dysfunction, c-Fos and NGF expression levels in the neuronal voiding centers, including vlPAG, were significantly increased with induced ICH, as compared to the control rats [125].

Various other structural defects may affect the PAG and lead to micturition problems. We here mention two case reports of subjects with neoplastic lesions or hydrocephalus, which compromised normal micturition by affecting the PAG. A lesion in the midbrain and upper pons was detected by MRI scan of a 34-year-old man presenting with a history of delayed ejaculation, voiding difficulties, and diplopia, which was a developmental abnormality such as a dermoid or teratoma [126]. In evaluation of patients with possible idiopathic normal-pressure hydrocephalus (iNPH) with typical imaging features (ventricular enlargement) and normal CSF pressure, a small bladder capacity and detrusor overactivity were seen in 95% of patients [127]. Since the PAG completely encircles the aqueduct, there is a possibility that this structure would be affected by minor dimensional changes in iNPH [127].

Sacral neuromodulation is commonly used in various neurologically mediated continence problems. The bladder filling and rest contrast, in eight spinal cord-injured (SCI) participants, elicited clear activation, measured by fMRI, in the PAG and in a continuous area in the right midbrain [106]. Following 2 weeks of pudendal stimulation treatment, abnormal PAG overactivity was decreased in all six participants, as well as in the four clinically improved subjects. Thus, the PAG may be overactive in the SCI group, following the sudden loss of the spinal afferent inputs [106]. The vlPAG overactivity in the SCI subjects was also demonstrated in a rat model, by increased expression of c-Fos or NGF, relative to the sham-operated group [128]. Abnormal PAG function would be restored to normal by sacral neuromodulation, in individuals with urinary retention [129].

### Functional Pathologies

These disorders encompass demyelinating diseases, PD, MSA, migraine, Wernicke’s encephalopathy, nocturnal enuresis, and urge incontinence, which will be elaborated below.

One of the most common demyelinating diseases is multiple sclerosis (MS), a common debilitating disorder with white matter plaques affecting any part of the brain. In 18.7% of MS patients, lesions were located in the PAG [130]. Thirty-six percent of these lesions were periventricular lesions of the third ventricle, extending toward the aqueduct. Bowel and bladder disability scores in MS patients are correlated with the volume of lesions in the medial frontal lobes, cerebellum, insula, dorsal midbrain including dorsal part of the PAG, and pons, areas known to be involved in the control of micturition [131]. As a clinical correlate, a 31-year-old man had suffered from sudden voiding difficulty and retention. A filling cystometrogram revealed an atonic bladder with diminished bladder sensation. Hyperintensities were shown in the PAG in T_2_W-MRI that were reduced after steroid therapy, with subsequent improvement of the voiding symptoms. He was suspected to have a demyelinating disease such as MS [132].

Patients with PD, which is an extrapyramidal disorder, may have lower urinary tract symptoms. Elimination of dopaminergic neurons by 6-OHDA microinjection into the PAG in a rat model of PD leads to altered micturition patterns [133]. Moreover, the reduction of the amplitude of the evoked potentials measured in the PAG, elicited by means of electrical stimulation of the pelvic nerve in the rat, is more pronounced in PD animals compared to sham animals, after the intravenous administration of an adenosine receptor antagonist [134]. Besides the direct effect dopaminergic lesions can have on the PAG, there is some evidence showing that the micturition problems in PD may be a consequence of a primary problem residing in the substantia nigra (SN), secondarily affecting the PAG by its projections toward the PAG. Increased c-Fos reactivity was observed in the PAG and ACC, in a PD rat model induced by 6-hydroxydopamine injection into SN [135]. Moreover, a 6-OHDA lesion in the SN produces a transient increase in voiding frequency within the first 2 weeks, with recovery of urinary function by 4 weeks post-lesion [136]. In PD patients, significant brain activation can be detected by PET in the PAG, during detrusor overactivity (measured by intravesical pressure monitoring) [137]. As another example of extrapyramidal disease, MSA, which includes micturition symptoms, shows neurochemical changes in the PAG (detailed above) [78].

Furthermore, a possible role of the PAG has been suggested in nocturnal enuresis. Single-unit activity in the lateral and ventrolateral columns of the PAG was linked to the occurrence of voids induced by continuous infusion of saline into the bladder of urethane-anesthetized rats, to mimic sleep-like brain states [55]. Almost a quarter of the recorded neurons were responsive during the micturition reflex. Their spontaneous firing rate in the absence of bladder stimuli decreased during slow-wave EEG states [55]. This suggests that the micturition reflex is reset centrally during the sleep. Failure of this mechanism could contribute to the development of nocturnal enuresis [138].

Various other functional disorders may have PAG malfunction together with micturition symptoms. Vegetative symptoms including increased micturition may occur in migraine [139]. More specifically, vlPAG was found in a PET study as a structure that may be implicated in migraine pathophysiology [140]. PAG dysfunction also has been shown in Wernicke’s encephalopathy [141]. A case report of Wernicke’s encephalopathy in a pregnant woman described decreased bladder volume and detrusor hyperreflexia in urodynamic studies. Brain MRI revealed abnormal intensities in medial thalamic-hypothalamic regions, and the PAG [141]. PAG [142] or midbrain [143] activation was shown by fMRI studies in urge incontinent patients as well. rCBF analyzed by PET is decreased in the midbrain during sacral neuromodulation in chronically implanted urge incontinent patients [144].

By its involvement in a multitude of disorders leading to micturition problems, the PAG must be included in future diagnostic or therapeutic algorithms concerning neurological causes of the bladder dysfunction. Specifically, with the availability of future high-precision MRI machines, the PAG can be more specifically evaluated, for diagnostic purposes.

## Conclusion

The PAG plays the role of a switchboard located in the brainstem, coordinating the evolutionary primitive and advanced brain centers. It has a broad spectrum of functions and has a paramount role in the control of micturition. The PAG functions as a sensory and motor relay station for the ascending afferents from the lower urinary tract, and descending afferents from the cortical areas. This is partly made possible by its special position at the intersection of the forebrain and the hindbrain. Two of the four columns of the PAG, namely the ventrolateral and dorsolateral columns, demonstrate more significant involvement in this respect. The vlPAG is more connected to the caudal structures, and the dlPAG is more connected to the cranial structures of the CNS [16]. The intercolumnar connections [11] traverse the information between vlPAG and dlPAG and thus complete a full circuit. Pathological conditions affecting the PAG may compromise the continence, and some of them may be detected by modern imaging techniques. Thus, the PAG will be a potential diagnostic and therapeutic target for specific incontinence problems and voiding dysfunctions. This may be done pharmacologically, by for example targeting its glutamatergic neurotransmission, or surgically, by DBS of particular PAG columns.
